# Proceedings of the 1^st^ Biennial Science of Global Prostate Cancer Disparities in Black Men

**DOI:** 10.1186/1750-9378-6-S2-I1

**Published:** 2011-09-23

**Authors:** Folakemi T Odedina, Vickie L Shavers, Richard Segal, Shannon Pressey

**Affiliations:** 1 University of Florida, College of Pharmacy, Gainesville, FL, USA; 2 National Cancer Institute, Division of Cancer Control and Population Sciences, Bethesda, MD, USA

## Introduction

Prostate cancer continues to be the most significant cancer proven to disproportionately overburden men of African descent, especially United States (US) Black men. Unfortunately, African American race/ethnicity is one of the three primary non-modifiable risk factors confirmed for prostate cancer.^1,2^ We are yet to confirm the primary reasons for the disparate burden of prostate cancer among Black men. This problem is further compounded by the fact that prostate cancer has become a major public health problem in developing countries. Thereby, global collaborations among prostate cancer scientists, clinicians, survivors and advocates are important to better understand the etiology of prostate cancer among at-risk Black men, and develop effective interventions to address these disparities.

The ***Science of Global Prostate Cancer Disparities in Black Men*** conference was organized to:

1. Provide opportunities for mutual learning, knowledge transfer, and collaborations among prostate cancer scientist clinicians, survivors and advocates.

2. Promote trans-disciplinary and multi-disciplinary prostate cancer research globally;

3. Facilitate networking among individuals involved in all aspects of prostate cancer control, education and research in Black men.

4. Facilitate the development of a global community of practice to address common challenges in prostate cancer, including prevention, detection, diagnosis, treatment and survivorship.

5. Contribute to a global impact against prostate cancer through research training and education programs for low-resource countries.

The primary goals for participants were to:

• Learn - Learn from internationally renowned speakers recognized in prostate cancer disparities and survivorship research.

• Discover - Discover the latest research findings on prostate cancer Prevention, Early Detection, Diagnosis, Treatment, Survivorship and End-of-Life.

• Connect - Connect with other Researchers, Clinicians, Patients, Advocates and Policy makers from North America, South America, Europe, Africa, and the Caribbean.

• Share - Share ideas with other conference delegates involved in all aspects of prostate cancer control and research in Black Men.

• Explore - Explore the world of trans-disciplinary prostate cancer research.

• Develop - Develop a global community of practice to address common challenges in prostate cancer disparities.

The 1^st^ Biennial Science of Global Prostate Cancer Disparities in Black Men Conference was held in the heart of downtown Jacksonville, Florida on August 27-29, 2010. A total of 96 delegates participated in the conference, including 21 patient/survivors, family, and friends. There were 15 international delegates from Africa, the Caribbean, and United Kingdom.

## Pre-conference workshops

There were a total of six pre-conference workshops, with three concurrent sessions in the morning and three concurrent sessions in the afternoon. Dr. Philipp Dahm chaired the first workshop. Dr. Dahm and Dr. Isaac Powell, presented the latest findings on ***Prostate Cancer Treatment & Outcomes***. The second workshop was chaired by Dr. Durado Brooks. Dr. Brooks and Dr. Kevin Stein discussed ***Prostate Cancer Prevention*, *Early Detection*, *and Survivorship***. Dr. Brian Cleaver chaired the third workshop and discussed ***Research Ethics: Protection of Human Subjects in Research***, a session mostly attended by international delegates. The afternoon concurrent workshops were chaired by Dr. Diane Woods, Dr. R. Renee Reams and Dr. Titilola Akinremi (Nigeria). The session chaired by Dr. Woods focused on ***Tools for Low Resource Countries***. She presented Community-based Participatory Research and Ms. Tara Hylton discussed Cancer Registration. For the fifth workshop, Dr. Reams (chair), Dr. Yehia Daaka and Dr. Jong Park presented ***Tools for Molecular Basis***. The last pre-conference workshop was on Research Collaboration with Low Resource Countries: Overcoming the Challenges, and was presented by Dr. Akinremi (chair) and Dr. Folakemi Odedina.

The ***Opening Plenary Session*** took place on Saturday, August 28 with opening remarks by Dr. Folakemi Odedina (Conference Chair) and program overview provided by Mr. Virgil Simons. The conference was officially opened by Senator Anthony Hill Sr (Minority Whip, FL). Dr. Vickie L.. Shavers (National Cancer Institute) and Col. Robert E. Porter (100 Black men Jacksonville) welcomed all conference delegates, and Dr. Paul Okunieff (UF Shands Cancer Center Director) gave the Welcome Address. The key note speaker was introduced by Dr. Rick Kittles (Scientific Program Committee Co-Chair).

A Community-Academic Town Hall Forum was held on Saturday, August 28 on ***PSA Testing in Black Men Across the Globe: Policy Implications***. This session was moderated by Dr. Charles Rosser and Mr. Anthony Grissett. The speakers/panelists were Dr. Frank Chinegwundoh ( UK), Dr. Femi Ogunbiyi (Nigeria), Dr. Robin Roberts (Bahamas) and Dr. Curtis Pettaway (USA).

## Keynote speaker

The key note speaker was Dr. John Carpten, PhD. Dr. Carpten is Professor and Director of Integrated Cancer Genomics Division, and Head of the Cancer Gene Discovery and Molecular Validation Unit at the Translational Genomics Research Institute. Dr. Carpten delivered an outstanding lecture on the status of the African American Hereditary Prostate Cancer Study Network. This multicenter collaboration has recruited a large number of African American families with men who have a high risk of developing prostate cancer to identify the genetic risk factors for prostate cancer.

## Bio-ecological system approach to understanding prostate cancer disparities

Nationally and internationally renowned experts were invited to present the latest findings on prostate cancer research for the conference plenary sessions. The first plenary session focused on ***Epidemiology and Genetics*** and was led by Dr. Rick Kittles who presented along with Dr. Tim Rebbeck. Mr. Theodies Mitchell, Jr. chaired plenary session two, with Dr. Alexander Asea, Dr. Richard Ablin, and Dr. Philipp Dahm presenting on ***Prevention and treatment***.

***Psychosocial and Behavioral Factors*** was the focus of plenary session three which was chaired by Dr. Carole Kimberlin, and included presentations from Dr. Folakemi Odedina, as well as Dr. V. Diane Woods. Dr. Rich Segal chaired plenary session four with presentations from Dr. Isaac Powell and Dr. Camille Ragin which focused on ***Geographical and Outcome Variations***.

Dr. Vickie L. Shavers chaired plenary session five with Dr. Rick Kittles, Dr. Femi Ogunbiyi (Nigeria), and Dr. Robin Roberts (Bahamas) presenting on ***The Translation Continuum: Innovative Research Approaches to Addressing Prostate Cancer Disparities from Bench to Bedside to Community***. Plenary session six focused on ***Funding Opportunities for Prostate Cancer Disparities***. The session was chaired by Dr. Frank Chinegwundoh (UK) with presentations by Dr. Alexis Bakos (National Cancer Institute), Dr. Carolyn Best (Department of Defense), and Dr. Ronilt Elk (American Cancer Society). The final plenary session was ***From Community to Bench: Researchable Community Issues on Prostate Cancer*** by Mr. Jim West (chair) and Dr. Willie Kimmons.

## Abstract presentations: podium and poster presentations

A total of 13 abstract submissions were accepted for presentation at the conference. Four posters were presented during a 3-hour poster session on Saturday afternoon. The poster abstracts were placed in either of two categories, ***Prevention & Early Detection or Prostate Cancer – Protective and Risk factors***. Dr. Richard Segal and Dr. Robin Roberts (Bahamas) moderated the poster sessions. The posters presentations were *Histopathological Review of Prostate Cancer in Lokoja*, *Nigeria* by Dr. Olabode Oluwole; *The Role of Dietary Fat in Prostate Cancer Risk in Jamaican Men: A Pilot Study* by Dr. Ayokunle Osho; *Correlation Between Gleason Grade And Age At Presentation Of Prostate Cancer in Black Men in Abeokuta*, *Nigeria* by Dr. A. Olutunde; and *Prostate Cancer: Histopathological Analysis of 66 cases* by Dr. Olabode Oluwole

There were nine oral podium presentations. Dr. B. Lee Green was the moderator for the first oral presentation session. The presenters were Dr. M. Tokunboh Odubanjo (Nigeria) who presented on the *Prognostic Factors for Prostatic Carcinoma Among Men in Lagos*, *Nigeria;* Dr. V. Diane Woods who discussed *African American Men’s Response to Prostate Cancer Prevention and Early Detection: The Pcap Toolkit*; and Dr. Kabore Aristide (Burkina Faso) who gave a presentation on *Prostate Cancer Outcome in Burkina Faso*.

The second oral presentation session was led by Dr. R. Renee Reams, who also gave an overview of *Detecting Gene-Gene Interactions in CaP Disease in African American*. During the same session, Ms. Jennifer Green presented a study on *The Efficacy and Toxicity of Selective Estrogen Receptor Modulators in the LNCaP Cell Line*; Dr. Rose Mary Stiffin provided an overview on *The Presence of Megakaryocytes in Cancerous Prostate Cancer Tissue*; and Dr. Hernan Flores-Rozas discussed the *Differential Contribution of Mismatch Repair Genes in the Processing of DNA Damage*.

Dr. Jong Park moderated the third oral presentation session, in which Dr. Titilola Akinremi (Nigeria) gave a presentation on the *Review of Prostate Cancer Research in Nigeria* and Dr. Belinda Morrison (Jamaica) gave an overview of her work on *Bone Mineral Density in Jamaican Men on Androgen Deprivation Therapy for Prostate Cancer*.

## The prostate cancer patient and provider symposium

The patient and provider symposium was organized by the Prostate Net and chaired by Mr. Virgil Simons, Founder and President of Prostate Net. This symposium was a unique experience allowing patients to learn with and learn from prostate cancer providers and researchers.

Patient/Survivor Track. The Patient/Survivor track was moderated by Dr. Brian Stone. He gave a presentation on the ***Prevention and Diagnosis of Prostate Cancer***. Following Dr. Stone’s presentation , a joint session on ***Prostate Cancer Treatment: What’s Best for You*** was led by Dr. Winston Tan, Dr. Stephen Ko, and Dr. David Theil. Dr. Paul Godley presented next on ***Efficacy of PSA Screening for Prostate Cancer among Elderly African Americans: A Case-Control Study***.

The Keynote Luncheon was given by Dr. Oliver Sartor who discussed the ***Emerging Trends*, *Issues & Treatment in Late-Stage Disease***. For the afternoon session, Dr. Brian Stone and Dr. Elisabeth Heath discussed the ***Treatment of Advanced Stage Disease*** followed by a presentation on ***Controlling Therapeutic Side Effects/Pain Management*** and by Dr. Stephen Ko and Dr. David Theil.

Provider Track. The provider track targeted clinicians and offered CME provided by the University of Florida. Dr. Stephen Ko and Dr. David Theil opened this track with a presentation on ***Managing Hormone Sensitive*, *Non-Metastatic Relapse Prostate Cancer***. The next presentation was ***Managing Castrate-Resistant*, *Metastatic Prostate Cancer*** by Dr. Winston Tan (chair) and Dr. Elisabeth Heath.

The Prostate Cancer Patient and Provider Symposium concluded with a Joint Provider-Patient Symposium session discussing the Future of Prostate Cancer Research and How to Address Clinical Trial Barriers. This session was moderated by Dr. Elisabeth Heath and Mr. Virgil Simons

## Special sessions

To foster dialogue between patients and leading prostate cancer clinicians, a ***Meet the Clinical Experts*** session was held on Saturday, August 28 presided by Dr. Christopher Williams. Six breakfast round table discussions were held on diverse prostate cancer topics led by Dr. Christopher Williams, Dr. Durado Brooks, Dr. Chidiebere Ogo (Nigeria), Dr. Adeteru Ayoade (Nigeria), Dr. Curtis Pettaway, and Dr. Charles Rosser.

## Future plans

Global collaborations are essential to better understand and effectively address the disproportionate burden of prostate cancer seen in Black men. To foster global collaborations and accelerate prostate cancer control and cure, the ***“Science of Global Prostate Cancer Disparities in Black Men”*** will be held biennially. The next conference will be in The Bahamas and will focus on the “Global Burden of Prostate Cancer: Economic, Clinical and Humanistic Outcomes of Prevention, Detection & Treatment”.
